# Sensorial pedagogies, hungry fat cells and the limits of nutritional health education

**DOI:** 10.1057/biosoc.2015.5

**Published:** 2015-04-27

**Authors:** Emilia Sanabria

**Affiliations:** a Ecole normale supérieure de Lyon, INSERM & Triangle UMR5206, Lyon 69007, France. E-mail: emilia.sanabria@ens-lyon.fr

**Keywords:** sensorial, obesity, feedback, endocrinology, health education, France

## Abstract

This article examines the way the category of ‘the sensorial' is mobilised across obesity research and care practices for overweight persons in France. The ‘natural' body is understood to have developed mechanisms that motivate eaters to seek out energy-dense foods, a hardwiring that is maladaptive in today's plethoric food environment. The article analyses the feedback models mobilised in scientific literature on the neuroendocrine processes regulating appetite. The analysis of how ‘the sensorial' is studied and used to treat patients provides a vantage point onto the ways foods and bodies transform each other. Recent findings show that fat cells influence metabolism by secreting hormones, revealing that eaters are affected by the materiality of the foods they ingest. ‘The sensorial' functions as a regulator in the feedback mechanisms where social norms regulating foodscapes become enfolded in the molecular processes that control appetite regulation. The article traces the work that the category of ‘the sensorial' does as it flows through the loops and feedbacks between scientific evidence, policy and care. It examines the way pleasure and the sensations of eaters are increasingly foregrounded in French nutritional health promotion strategies in a context where informing eaters is increasingly deemed ineffective.

## Introduction

Valérie, 48, has gone on and off diets for over 30 years and has what she describes as a terrible relationship to her body. She recently calculated her body mass index (BMI) using an online calculator (www.imc.fr). At 79 kg for 1.77 m she has a BMI of 28 and is classified as overweight. When she entered her measurements, a flashing red dialogue box appeared on her screen, warning her, “ATTENTION: you are OVERWEIGHT. Consult your doctor or a nutritionist immediately to determine the causes. You must lose weight”. Valérie has consulted innumerable doctors about her weight and tried most of the mainstream weight loss diets. Whatever weight she has lost, she has always gained back, sometimes in excess. As summer approaches, she makes an appointment at her local hospital's endocrinology service and a few tests later meets with an endocrinologist who enters her into an ‘eating rehab' programme. “You have a slow metabolism”, he tells her. “You're unlucky, you gain weight just by looking at food. It's not fair, but it's your reality. Your ancestors were famine survivors and have passed on these genes to you. It's not your fault. But you'll need to change the way you eat if you want to lose weight durably”.

This article is based on ethnographic work with nutritionists who believe that long-term effective weight loss is only possible if you eat ‘to feed pleasure'. This group of practitioners does not forbid any kind of food – not even greasy chips or chocolate mousse – and even teaches patients the art of food *dégustation*. Patients who have endured decades of restrictive diets are invited to taste and savour *with* their dietician the very foods that have been proscribed and banished but which they feel they irremediably succumb to. The GROS (literally fat/big), or Research Group on Overweight and Obesity, is an association of health professionals comprising nutritionists, dieticians, psychiatrists and psychologists working with overweight and obese patients in reference centres or private practices across France. The network counts nearly 200 specialists across France trained in the GROS method and organises a yearly conference and training programme (which I have partially undertaken). The GROS method is founded on a programme of therapeutic education that is centred on the eater's bodily sensations. Pleasure is a leitmotif.^1^ The GROS approach takes as its starting point the idea that food intake is regulated by complex sensorial mechanisms that signal to the eater when to initiate or end a meal. GROS practitioners consider that all dietary recommendations, including those promoted by the French National Program for Nutrition and Heath (PNNS), encourage eaters to “eat with their head rather than with their sensations,” as GROS co-founder Gerard Apfeldorfer summarised in an interview. The GROS' explicit goal is to reunite eaters with the pleasure and the sensations of eating. Rather than ‘cognitive messages' or dietary advice, the GROS considers that ‘sensations' should guide eating. The GROS website's “regulating alimentary behaviour” information pages asks, “Why tell the French population how they should eat, when every human being is naturally equipped with a system of sensorial regulation that enables him or her to eat adequately?”

As in most European nations, vast public health initiatives were launched in France to tackle non-communicable, chronic disease associated with high BMIs. While the causes, mechanisms and means of calculating the rising rates of obesity are contested and uncertain, emphasis is recurrently placed on the shift between energy intake and expenditure in societies where energy-dense foods have become widely available and aggressively advertised. Given this context of uncertainty concerning how to qualify and circumscribe the ‘problem', let alone define its aetiology, developing effective public health responses presents considerable challenges. This article traces some of the shifts in the epistemological understanding of appetite regulation and examines how these are mobilised to different care or policy ends. It aims to contrast two positions and map an emergent transformation in understandings of obesity and its prevention. The first centres on informing eaters about nutrients and on proscribing certain calorie-rich foods. The second, more emergent, is the position developed by practitioners such as those trained in the GROS method and builds on scientific understandings of the role of sensorial processes in appetite regulation. It constructs its rationale on an explicit rejection of dieting practices, noting that diets ultimately fail to bring about weight reduction and lead to overall weight gain or disordered eating in the long term.^2^

I examine the uncertain materialities these sensorial pedagogies bring to life and trace the boundaries where foods and eaters, and matter and subjects, merge and transform one another. By considering how the appetite control systems of eaters are conceptualised and presented as undermined by a contemporary environment of irresistible highly palatable foods, my aim is to examine how the promotion of conscious and intimate sensory engagements with foods is understood by practitioners such as those working with the GROS method to provide an alternative response. In so doing, I examine the ways in which scientific evidence concerning appetite regulation, taste, pleasure and sensoriality is mobilised to explain the purportedly epidemic rise in obesity. What emerges is the idea of a misfit or maladaptation between the plethoric environment in which ‘we' live today and the body ‘we' are said to have inherited from a distant Palaeolithic past whose regulation system is geared towards defending its fat stores.

The analysis of how sensorial mechanisms are studied (in scientific practice) and used to treat patients or to promote health (in clinical practice or health education) provides an interesting vantage point into the ways the materialities of foods and bodies mutually transform each other. The sensorial systems that regulate food intake are triggered, at one end, by materials (foods), information about which is gathered through olfaction and sight and through taste and somesthesia,^3^ once the food has entered the eater's mouth (although olfaction continues and somesthesia may begin earlier).^4^ Bodies and foods become so profoundly enfolded into each other that it is impossible to determine where one begins and the other ends. The cascade of metabolic processes that takes place in bodies once food has triggered taste and olfaction, and the complex hormonal feedback mechanisms that follow, leads across a threshold of sorts. As endocrine processes spark off neurological responses, the tangibility and materiality of the process becomes more uncertain. The relation between the body's sensorial apparatus, the environment, and the relative importance of cognitive and automatic processes in food intake regulation forms an important area of research in ‘obesity science'. The findings developed in this area feed a host of popular science books and media coverage on topics such as food addiction, willpower, dieting and overeating. They are in turn picked up by actors in the field of food health promotion such as those I have been working with. The questions that are being formulated by clinical and biomedical research around the topic of the sensorial concern the biological mechanisms that are driving people worldwide to eat more, and more of certain kinds of foods.

My aim is to attend to the way the category of the sensorial is used in the practices put in place by the GROS (and similar groups outside France). I start by contextualising their approach in a public health landscape dominated by health information and education. I then analyse two key areas of scientific research that the GROS draws on to found its rationale: the analysis of the active endocrine role of fat tissue in the regulation of appetite, and debates surrounding the feedback models that regulate energy in the body. Here, I draw on the distinction between first- and second-order cybernetics to examine the way in which homoeostatic regulation is repeatedly set against hedonism in discussions of obesity. Are the hedonic processes that are said to be disrupting homoeostatic regulation of appetite merely a dis-regulation in a closed loop system or a different kind of system altogether? How might these conceptual differences map onto the debates taking place around regulation in obesity science? The complex models of homoeostasis and analyses of the mechanisms of its contemporary dis-regulation that circulate in this field open up important questions for public intervention and the individualisation of responsibility for health, and invite us to reconsider the ways in which nutritional recommendations cast specific relationships between eaters and the foods they consume.

## Beyond Informing: Forging the Domain of the Sensorial

Mol (2012, p. 379) notes that “the overriding message of most dieting advice is that a person who wants to lose weight needs to overrule the desires of her craving body”. The fact that people are taught to relate to food in a calculative way suggests, she argues, a particular enactment of a mind-body distinction according to which without information, bodies would overeat, indulging in pleasure. The “ontonorms” at play in the Dutch dieting advice she analyses construct the natural homoeostatic capacities of bodies as an ideal and “hedonistic enjoyment” as a danger (Mol, 2012, p. 383). Information on calories, nutritional content of foods and the importance of a balanced diet is presumed in the dietary advice provided through public health nutrition programmes to enable “the rational mind to take control of the pleasure seeking body,” Mol notes. The GROS explicitly posit that information and rational (or “cognitive,” in their terms) control not only fail to bring about durable weight loss but actually impair eaters' natural bodily capacities to regulate weight. Like the practitioners on the margins of Dutch dieting that Mol describes, the GROS – who have developed a more structured and centralised approach than their Dutch colleagues, encourage people to taste, savour and *enjoy* their food.

Much of the debate in the obesity arena concerns the relative importance of willpower and biological factors in the development of adiposity and its control. This debate goes back to the early part of the twentieth century and to the shifting influence that endocrinologists and psychiatrists had in defining the aetiology and outlining treatment for obesity. Rasmussen (2012) traces the shift in the United States from an endocrinologically oriented approach that centred on glandular disorders to a psychiatrically oriented one that focused on the neural determinants of excess appetite. Obesity was framed as an addiction and constructed as a disorder of willpower (Rasmussen, 2012, p. 890). Interestingly, a new current in obesity research is attempting to re-qualify overeating as a form of addiction. However, this can be seen as an attempt to map public policy responses to BigFood on those that have been carried out against BigTobacco (see in particular Brownell and Gold, 2012). So while food addiction is gaining renewed attention, the rationale behind this is to shift the blame from individuals to industry-related activities that deliberately harm health through what the Lancet Non-Communicable Disease Action Group refer to as “corporate disease vectors” (Moodie *et al*, 2013). It is beyond the scope of the current article to examine why educating eaters and conveying information about nutritional and calorific content of foods emerged as a key domain of intervention in nutrition. Suffice it to say that nutritional health education is increasingly deemed to be at best a weak strategy in the face of the strong biological drive inscribed in our hardwiring to seek out calorie-dense foods and at worst a harmful or counter-productive one, shifting blame onto individuals (James, 2007; Herrick, 2009; Guthman, 2011). Part of the problem lies in the fact that the epidemiological patterns of obesity are contested (Gard, 2011), as are the causes and mechanisms that lead to the widely denounced gain in girth. In the absence of effective, population-wide interventions (such as pharmaceutical magic bullets or surgical solutions), information-education-communication models of behaviour change have thus been favoured.

The PNNS, French National Program for Nutrition and Heath (Ministère de la santé, 2011, p. 10), adopts a broad definition of ‘nutrition' that includes physical activity and recognition that nutrition includes “nutrients, foods, social determinants, cultural, economic, sensorial and cognitive” aspects of food behaviours. Nevertheless, the PNNS identifies information, communication and education to shape food behaviours and physical activity as its main ‘strategic lever'. The idea that obesity would be solved if people ate balanced meals and were less sedentary remains a tacitly shared one – largely reinforced by the tenor of health promotion messages. One implication of such a view is that if people are informed and fail to change their behaviour they are seen to be individually responsible for any ill health that may ensue. The relationship between access to information and ‘positive' health behaviours has been problematized in a number of ways in public health, notably through greater attention to the social determinants of health (CSDH, 2008) or to the complex interplay of environmental, urban and metabolic factors in obesity. The Foresight Report on obesity put forward the notion of “passive obesity” to suggest that default choices posed by the social and built environment mean that combatting weight gain requires active strategies.

People in the UK today don't have less willpower and are not more gluttonous than previous generations. Nor is their biology significantly different to that of their forefathers. Society, however, has radically altered over the past five decades, with major changes in work patterns, transport, food production and food sales. These changes have exposed an underlying biological tendency, possessed by many people, to both put on weight and retain it. (Butland *et al*, 2007, p. 5)

At a recent conference on the social determinants of health held in France, one economist even suggested that health education had deepened health inequalities as more educated segments of the population had benefited disproportionally, deepening the gap in health outcomes across the social economic gradient. The relationship between providing information through public health campaigns and changed behaviour has also been problematized by behavioural economists who are paying increasing attention to the ‘non-rational' processes that shape health decision making. The concept of nudging (Thaler and Sunstein, 2008), itself closely tied to social marketing, draws on a distinction between reflective and impulsive decision-making processes. Nudging opposes rational, reflective or cognitive processes to ‘automatic, affective' reactions and ‘feelings' that are triggered by environmental cues and driven by the lure of pleasure. In the context of a generalised moral panic surrounding rising rates of obesity, disparate approaches such as those appropriated by some eating disorder specialists adopting cognitive behavioural or mindfulness therapies (for example, Kristeller and Wolever, 2011) or organisations such as SlowFood that have taken up taste education (Barzanò and Fossi, 2010) have coalesced around the questioning of nutritional interventions that address overeating through abstract, disembodied information.^5^ The rational, autonomous individual who has been the target of public health initiatives is attacked, so to speak, on two fronts by the emergence of a critique of health information and communication (Gastaldo, 1997; Evans *et al*, 2008; Whithead and Irvine, 2011; Ayo, 2012; Fitzpatrick and Tinning, 2014). On the first, she is seen to be fighting against an obesogenic environment and structural forces that determine she will have disproportionally worse health outcomes if she is female and of low social economic status (in France, and where obesity is concerned). On the second, she is seen as driven by unconscious, affective and automatic – largely biologised – forces that determine her behaviour and actions against her will or rational intentions (Thaler and Sunstein, 2008; Rice, 2013).

The GROS, for its part, draws a fairly radical distinction between what they refer to as “cognitive” or informational approaches and those, such as theirs, that centre on *les sensations alimentaires* (alimentary sensations). The ‘sensorial' emerges as a central category in their practices. While there is an important literature on the anthropology of the senses (Serres, 1985; Classen, 1993; Seremetakis, 1994; Korsemeyer, 2002; Howes, 2003, 2005) and on the importance of attending to the senses in the study of health and illness or eating (Hinton *et al*, 2008; Nichter, 2008; Sutton, 2010), less has been written on how the sensorial may itself be rendered a therapeutic tool. The GROS practices I examine participate in what Seremetakis (1994, p. 123)^6^ has referred to as a modern concern with “sensory loss”. These practices aim to “re-sensitise” eaters who have become “divorced” from their sensations. They aim to bring heightened attention to the senses and the differences in sensory input (differentiating textures, olfactory, gustative or visual stimuli) and the importance of sensorial blending (sensations that are produced by the interaction of multiple sensory inputs). Nichter (2008) notes that meaning and experience of sensation are shifting and deeply biosocial (p. 186). He draws links between the increasing medicalisation and pharmaceuticalization of bodily sensations and the decreasing tolerance to discomfort and sensation. Sensations are so numerous in our daily lives that only some of them are cognitively processed and given meaning. They are typical hybrids and as such provide a zone through which biologies are encultured. As certain sensations become more salient through being stimulated or made culturally meaningful, certain neural pathways are reinforced, thus increasing the “inducibility” of the sensation, a process that Hinton *et al* (2008) refer to as the kindling of sensation.

Kindling aptly describes the industrial processing of “hyperpalatable foods”, or foods that GROS practitioners refer to as “highly sensorial.” Palatability is the fine-tuning of foods to enthral the senses, stimulate appetites, drive wanting, override satiety and motivate eaters to pursue more of that taste. In *The End of Overeating: Taking Control of Our Insatiable Appetite*, ex-FDA commissioner, paediatrician and Dean of Yale medical school Kessler (2009) argues that the multisensory dimensions of hyperpalatable foods reinforce reward (p. 49) and are drivers in overeating (p. 98). “Mixture is where the magic happens,” a food industry consultant told Kessler (2009, p. 100). Industry, Kessler tells us, is shifting towards greater complexity, combining optimal amounts of sugar and fat to attain a “bliss point” and developing a versatile range of “inclusion products” to add crunch, blasts of flavour, and dynamic contrasts in flavour or colour, and to enhance the sensory properties of foods that drive desires. Processing makes chewing increasingly unnecessary, facilitating swallowing and reducing the time food spends in the mouth, thereby undermining satiety mechanisms that arise when food is savoured and not just gobbled down. In what follows I attend to the way the deeply encultured category of ‘the sensorial' is increasingly being mobilised across a range of care and prevention practices in the field of nutrition. I present some of the scientific evidence that is drawn upon in this context with specific reference to the practices deployed by the GROS, and the relations that they draw between sensorial overriding and overeating.

## Returning to Alimentary Sensations

Cognitive restriction is a central concept for GROS practitioners. Initially developed in 1975, the term is reworked by Polivy and Herman (1991) to refer to eating that ceases to be controlled by internal factors and alimentary sensations and that becomes controlled and planned according to cognitive factors such as dietary prescriptions. Many of the obese patients that GROS practitioners treat in their private practices have been through years of diets that GROS practitioners feel have led them to lose touch with their bodily sensations. The aim of treatment is to shift from a mode of eating that is cognitively determined (that is, by external injunctions and beliefs) to one that is “intuitive and *sensorial*, governed by an attention to and a respect of alimentary sensations and emotions” (Zermati and Apfeldorfer, 2010, p. 154, my translation).

During a GROS training session I attended in Spring 2012 along with two dozen psychiatrists and dieticians from across France, GROS co-founder Zermati spoke of a diet as any form of “deliberate control of food intake on the basis of external, cognitive factors that take precedence over internal signals such as those that ensure our energetic homeostasis”. He then provided a detailed explanation of why, on the basis of the scientific evidence we have on adipose tissue hyperplasia and negative feedback, people cannot lose weight efficiently or durably, let alone happily, when they use cognitive restraint “against” their bodies. Apfeldorfer, Zermati's colleague and co-founder of the GROS, explained the GROS method to me in the following terms:

Our neurophysiological needs impose themselves upon us through a series of sensorial mechanisms. Breathing is vital and independent of our control. It is an automatically regulated processes. Eating like sleeping is a semi-automatically regulated behaviour. If we are tired we can resist sleep, but the more we stray from our sensations, the stronger the backlash. Because food was not always available in our past, we have developed mechanisms to tell us when to seek out and end meals as well as to stock calories. Sometimes we have to make do with the sensation of hunger, and at others to eat without hunger. Pleasure was once the regulator, but because our pleasure centres are overstimulated by hypersensorial foods, we have lost track of our internal messaging system. The GROS defends an approach based on the attention to and the respect of bodily sensations through work on the events that impede us from respecting these sensations and which leads to addiction-type processes.

The GROS method is put to work in a range of therapeutic settings, from private dietetic practices to clinics specialising in the treatment of obese patients. Founded in 1997, the GROS counts nearly 200 practicing members across France who have received certification. The typical GROS patient is female, has been on restrictive diets all her life and consults in a private practice through the liberal system of ‘town' (as opposed to hospital) medicine. However, a growing number of specialised services across public hospitals and private clinics are training their staff in the GROS method and integrating aspects of the sensorial approach into the care practices they offer their patients. The method itself centres on a series of practical exercises and techniques that include experiments with taste. Many practitioners begin with a *dégustation* of a ‘problem food'. Patients are invited to bring in a food that they cannot resist and that they readily overeat. This invitation to explore and consume a taboo food with a dietician is also a means of building trust with the patient and is used by GROS practitioners to demarcate their approach from all the ‘restrictive' injunctions patients have often endured previously. Drawing on the model of a wine tasting, patients are invited to consciously examine the food item they have chosen, its shape, texture or smell, before beginning the *dégustation*. Each mouthful is taken ‘in full consciousness' of the cascade of sensations that unfold as the dietician explains the molecular and physiological processes that produce a taste and through which the organoleptic properties of the food being consumed are relayed into a mental image and a sensation of pleasure. As the *dégustation* proceeds, the patient is invited to note changes in texture and sensation. Is the pleasure experienced in the same way? Does it peak or drop? Does the food item taste the same after the *n*th bite? Such an experience is expected to bring awareness to the natural internal signalling mechanisms that the ‘disregulated' eaters ‘we' have become fail to attend to. The objective is to find the point of *rassasiement*^7^ at which the food no longer has the same taste and at which both the wanting and liking of it decrease. Although the awareness that is sought through the body is set against that derived from rational, mind-driven control, it is interesting to note the way terms such as ‘mental images' or ‘full consciousness' creep back in.

The GROS training introduces practitioners to an explicitly cybernetic understanding of appetite regulation. As Zermati explained during a training session, when the homoeostatic system is internally unbalanced, a biological value (such as a drop in blood sugar) is transformed into a specific sensorial message. When this message is transformed into a sensation, it activates a behaviour such as the initiation of a meal or a desire for a specific food. When the need has been satisfied, a sensorial message in the form of the feeling of satiety is returned to end the meal. Negative feedback characterises a balanced homeostatically regulated system, where the need diminishes with the behaviour. In a ‘dis-regulated' system, the need (hunger) augments with the activity: there is positive feedback. Satiety cannot be experienced if eating takes place too fast, or while focusing attention on another activity simultaneously. When foods are enjoyed and not just gulped down, chemically induced gustative modifications occur, triggering sensory-specific satiety. Zermati and Apfeldorfer (2010) ask how one can understand one's hunger and sensations if one is plugged into the outside world, eating unconsciously and without truly stopping to feel one's internal sensations.

GROS-led experiences also include work on the emotional aspects of hunger. During their training, dieticians and health professionals seeking GROS certification are themselves invited to experiment with the sensations and emotions raised by hunger through exercises that include skipping meals and recording the physical and emotional sensations this gives rise to. Ensuing discussions during training provide a platform from which to introduce the scientific literature on which the GROS founds its rationale. Hunger is graduated and the emotions it gives rise to (such as anxiety, fear, anger, sadness) identified. The experiment aims to rid the sensation of hunger of any anxiogenic emotions it may carry and return it to its primary physiological function of indicating a need to feed. Patients may also be invited to keep a *carnet des sensations* (sensations notebook) where all foods consumed in a week are noted to bring attention to the temporal and emotional contexts of eating. This teaches patients to differentiate eating that is triggered by hunger from eating that is triggered by emotional states. It leads them to identify the emotional states that lead to a desire to eat. As Zermati and Apfeldorfer note (2010, p. 138), contrary to common understandings, it is not hunger that makes us eat but the motivation to seek out food that results from hunger. This may also result from other factors such as emotional states. One of the fundamental issues in GROS therapeutic practice is learning to distinguish between desire to eat that is triggered by hunger and one that is triggered by an emotion. Blame and guilt that result from a failure to follow dietary recommendations are seen by GROS practitioners – and by others approaching eating in a similar vein – as having an embodied effect and increasing the feelings of hunger, decreasing the feeling of satiety and satisfaction, and putting the body in ‘starvation' mode, hence increasing the likelihood of stocking the calories ingested. The sensorial thus becomes an interface between processes that are classified on either side of body/mind or biological/social distinctions. Satiety, in this model, and the stabilising of the energy balance are dependent on guilt-free pleasure.

Zermati and Apfeldorfer (2010, p. 137) note that obesity is due to two consecutive dis-regulations. The first is a dis-regulation of the system of homoeostatic regulation that leads eaters to consume more than their calorific needs, due to the fact that they are no longer sensitive to the natural sensorial mechanisms that signal satiety. The second is linked to what they refer to as the “set point”, which is a function of adipose tissue signalling and which is determined by the quantity of adipocytes in the body. As these multiply they raise the set point, making weight loss beyond a certain threshold practically impossible. For these reasons, dieting cannot lead to durable weight loss because the augmentation in adipose tissue is sometimes not reversible, and mental control over alimentary behaviour cannot be maintained in the long run. In the remainder of the article, I thus draw on two key issues that arise from the scientific debate that the scientific committee of GROS draws on: the shift from homoeostasis to hedonism and the role of adipose tissue in the regulation of appetite.

## The Agency of Fat: Adiposity and Negative Feedback in Plethoric Environments

This section focuses on some of the scientific work being done in the area of food intake regulation and examines the disquieting effects foods are presented as having on eaters in some of this literature. This raises specific questions for the issues I opened with concerning preventative or educational strategies. If foods exert their influence on eaters in ways that bypass or circumvent eaters' wills, what is the effect of educating eaters about food groups, or the importance of eating five fruit and vegetables? The regulation of human appetite is controlled by complex feedback mechanisms involving a range of hormones that connect the different sensory organs such as the tongue, taste buds and gut to nodes in the central nervous system such as the hypothalamus. These elaborate pathways are still being explored as new hormones and receptors are discovered and their functioning explained. In 1994, the hormone leptin was discovered and shown to have a central role in appetite regulation. Leptin is produced in the adipose tissue and plays a key role in the regulation of hunger. Two key mechanisms are at play in appetite control and food intake regulation: a homoeostatic mechanism and a hedonic one. Much of the current work being carried out in this area points to the increased role of the latter in the contemporary food-rich environment to which we are increasingly exposed. The argument is that the marketing by BigFood of energy-dense hyperpalatable foods stimulates the hedonic system, driving wanting and exciting pleasure, thus overriding homoeostatic control mechanisms (Kessler, 2009; Gearhardt *et al*, 2011; Brownell and Gold, 2012).

But first let us briefly consider how the homoeostatic system is said to function. Energy homoeostasis works to maintain the stability of body fat stores by regulating both food intake (the motivation to initiate and end meals) and energy expenditure (the rate at which calories are burnt). In their conclusion to a review on central nervous system food intake control published in *Nature*, Morton *et al*, 2006, note that there is no consensus on the fundamental aspects of obesity parthogenesis. The review examines evidence concerning the molecular and behavioural mechanisms that link modulations in body fat to the regulation of food intake through mechanisms such as satiation and motivation to initiate meals. It shows that central nervous system-controlled energy homoeostasis responds to changes in body fat stores. As these stores diminish (as weight is lost) “the motivation to find food and the size of individual meals tend to increase until energy stores are replenished” (Morton *et al*, 2006, p. 289). Adipose negativity feedback signalling posits that signals inform the brain of changes in body fat stores, leading to adaptive adjustments that aim to stabilise fat stores by acting on the regulation of satiety perception and brain reward circuitry. The authors thus make a claim that goes against commonly held assumptions about weight gain, proposing that “obesity involves the *defence* of an elevated body weight, rather than the absence of regulation”, and that the “global obesity pandemic” (*sic*.) is the product of “deleterious interactions between obesity-promoting environmental factors and homeostatic control systems”. This to say that the homoeostatic regulatory mechanism is driven to defend against fat loss rather than to prevent weight gain. This is often explained by reference to our palaeontological past, in which periods of feast and famine were common and where the capacity to maintain fat stores would have been an evolutionary advantage. In this sense, the ‘natural' body ‘we' are said to have inherited from this collective past has mechanisms that motivate eaters to seek out energy-dense foods, a hardwiring that is maladaptive in today's plethoric environment. The adaptations that are imagined to have favoured the survival of our ancestors, such as mechanisms to maintain fat stores in prevision of periods of famine, are understood to be our modern plagues, leading to the increase of cardiovascular disease and type-2 diabetes.

Kessler (2009) argues that the homoeostatic regulation system described above is often overridden by another mechanism with different brain circuitry. “This is known as the reward system. And […] in the fight between energy balance and reward, the reward system is winning”. The reward system reinforces motivation to seek out certain desirable foods. It is based on a feedback system that teaches eaters to associate specific calorie intake with particular tastes and foods. This is an acquired process, built up over time and one that is reinforced by the increasing presence of hyperpalatable foods in global foodscapes. In a sense it can be argued that the turn to the sensorial dimensions of appetite regulation in obesity prevention circles arises subsequent to the close attention given by the food industry to tailored modifications in the sensorial qualities of processed foods. Guyenet and Schwartz (2012) define reward as “the process whereby certain behaviours are reinforced in response to specific environmental stimuli.” Palatability, they argue, has been consistently shown to influence meal size in humans. As Guyenet (2012) notes in a blog post, alluding once more to an evolutionary explanation:

Our brains are highly attuned to these qualities because they're all elements of nutritious, calorie-dense foods that would have sustained our ancestors in a natural environment, but today, the exaggerated combinations of these qualities used by processed food manufacturers […] overstimulate our natural reward pathways. Commercial foods are professionally designed to maximize reward, because reward is precisely what keeps you coming back for more.

These developments, and the shifts in language that are required to communicate them, are interesting in that they give adipocytes substantial agency. These cells are said to actively defend the fat stores in bodies through a range of metabolic, neurologic and endocrine processes. They are, as it were, hungry, and in turn make eaters hungry. Eaters' capacities to make rational choices are subverted by these hungry fat cells. They decide for them, or interfere with their decision-making capabilities. What does it imply to say that it is the fat that is hungry, rather than the eater?

In its training programme the GROS gives considerable attention to recent findings on the mechanisms of adipose tissue growth. They regularly refer to the fact that there are two mechanisms of adipose tissue development: adipocyte hypertrophy and adipocyte hyperplasia. In the first case, fat cells increase in size as they stock excess energy. This mechanism of weight gain is easier to fight as weight is lost through the ‘emptying out' of adipocytes. However, recent attention has turned to a second process of fat accumulation that consists in the multiplication of fat cells (Jo *et al*, 2009). There is debate concerning whether or not adipocyte hyperplasia occurs after the age of 20 (Tchoukalova *et al*, 2010). What is significant for the present purposes is that the adipose tissue has come to be portrayed in such literature as having an active endocrine function. It is now conceived of as “an organ in and of itself, like the pancreas or liver,” as one nutritionist put it to me during the 2012 GROS congress. Attention is being given to the role of fat cell progenitors from different parts of the body in generating adipose tissue with different inflammatory or ‘dis-regulatory' impacts. While hypertrophied adipocytes can be expunged, their multiplication at a young age implies that the future adult will have a much greater susceptibility to weight gain due to the fact that the set point defended by negative feedback is higher.

The relation between fat and appetite as it is described in scientific investigations into the endocrine functions of adipose tissues and the active role fat tissue plays in the regulation of appetites is a classic case of what Leslie Aiello, President of the Wenner-Gren Foundation for Anthropological Research, has termed a “paleofantasy”. Paleofantasies are stories about human behaviour that are constructed upon evolutionary narratives based on limited fossil evidence. In an essay citing Aiello, evolutionary biologist Zuk (2009) notes that “The notion that there was a time of perfect adaptation, from which we've now deviated, is a caricature of the way evolution works.” The idea of a mismatch between the body we have inherited and the environment we now live in is so recurrent it appears self-evident. The recursive nature of the argument gives weight to the idea that the complex molecular pathways that regulate our appetite have developed in response to a poor, famine-ridden environment. It is as if the environment we evolved in during the Pleistone were now irremediably enfolded in our molecular make-up. This “primordial gluttony”, as Kluger (2007) puts it in his review *The Science of Appetite*, arises from a prehistoric time when we were “programmed” to overeat. In this imagined race for survival, those who did not lose their appetite immediately put on more weight and survived the next famine, passing on the genes.^8^ Yet, along with many public heath nutritionists, I would like to suggest that this mismatch may not be so accidental, as it were. The shape urban environments are taking worldwide, interspersed as they increasingly are with a dense network of outlets for highly processed and highly palatable foods available at all hours of the day and night at prices defying competition, are the product of specific forces that have tailored their marketing strategies to certain features of ‘our' inherited hardwiring (Hawkes and Buse, 2011; Monteiro *et al*, 2011; Kleiman et al, 2012; Stuckler and Nestle, 2012; Stuckler *et al*, 2012).

Hedonic and homoeostatic mechanisms are deeply intermingled. As excessively rewarding foods are consumed, body fat stores increase, and with them, the level of body fat that is ‘defended' by homoeostatic feedback mechanisms. This raises important questions concerning the burden of responsibility that is placed upon eaters, particularly overweight or obese eaters. What are the implications of recognising that adipose tissue stimulates appetites? Can this move us beyond the responsibility placed on individuals to care for their own health despite living in environments widely recognised to be deleterious to health? Or does this on the contrary further render overweight people targets of specific risk reduction action? As far as the GROS approach is concerned, the objective is explicitly to *déculpabiliser* (de-blame) overweight people and to demystify commonly held ideas concerning the fact that they eat more (which may not be true) by demonstrating that their bodies are defending a larger body fat store (which does not necessarily imply higher food intake). The objective of training practitioners on topics such as adipose tissue negative feedback or adipose hyperplasia is not so much to biologise behaviour as to reveal that the standard treatment – cognitive dietary restraint – is inefficient and profoundly damaging for many patients. If we consider, as the GROS suggests, that the body naturally strives to get back to its set point, as defined in part by levels of adiposity, and that this set point cannot be modified (without surgical intervention, but even then there is controversy), then dietary restraint is a very limited response.

## Feedback Models and Complex Systems in Changing Environments

Set point, negative feedback and homoeostasis are all terms that have been borrowed from cybernetics and the science of complex systems regulation. As we have seen, there is an extensive scientific literature that examines the way hunger and satiety are regulated. Such processes are described as being regulated by complex neuroendocrine processes that involve a number of brain centres (some of which are even being located in the digestive system), sensory receptors throughout the body and a list of hormones that continues to expand. Body weight is modulated by interrelated systems that function on different temporalities, a shorter one modulating blood sugar levels and a slower one modulating adiposity. Feedback mechanisms maintain stability in changing environments, and are thus seen as playing a vital evolutionary role as we have seen here. However, as we have seen here, a growing number of obesity researchers are calling attention to the fact that environmental conditions have shifted so drastically with the widespread availability of calorie-dense foods that negative feedback mechanisms are failing, generating pathology.

The sensorial approach defended by GROS raises important questions concerning the complex feedback relations between the commensal, social and environmental dimensions of eating that shape the sensorial mechanisms through which people gauge hunger and satiety.^9^ Social norms concerning appropriate meal frequency, or portion size and environmental factors concerning availability of foods in the various environments people circulate through, become enfolded in the molecular processes that control appetites. For example, hunger signals are known to occur at regular intervals in accordance with habitual meal schedules. Nichter (2008, p. 173) notes that the rural agriculturalists he worked with in India literally told the time of day by their rumbling bellies. The sensorial domain is thus socially modulated in complex ways. Although advocates of sensorial education make a strong distinction between ‘external' eating cues and ‘internal' sensations, the demarcation is not so clear if we consider the extent to which internal sensations respond according to these elaborate feedback mechanisms to external, societal, conditions. How, then, can we effectively return to our bodily signals in a context where these are always already shaped by the social context that is understood to disrupt our natural capacity to self-regulate? There is a reflexive loop here that poses considerable difficulties to practitioners and patients alike, as attested by the centrality of the task of learning how to differentiate between ‘real' physiological hunger and ‘emotional' or ‘triggered' hunger. Such issues, for example, took on a particular difficulty in the context of discussions in the GROS training sessions I attended and in debates following GROS method presentations at conferences, concerning child feeding. While children are often taken as a prime example of our innate capacity to eat in accordance with our energetic needs (and not with emotions or as driven by hedonic processes), the importance of socialising children's senses is emphasised as, without guidance, it is suggested they would eat only sweet and calorie-dense foods. These contradictions point to the subtle and fragile nature of the feedback mechanisms that regulate appetite. I would like to suggest that the conceptual difficulties raised through such discussions point to the ways in which bodies and their environments are tacitly classified on different ends of a polarity. The sensorial mechanisms these different practices seek to engage are interesting precisely in that they straddle these conceptual divides between the inside and the outside of bodies, between matter and subjects, between conditioned and inherited processes.

Feedback models are central to the way endocrinologists are trained to think about the regulation of the energy balance. Complex systems-thinking is increasingly important to analyses of obesity policy (Ulijaszek, forthcoming). However, systems thinking is itself a complex and evolving field. I briefly turn to some important distinctions between models of complexity and consider their implications for the ways in which the complexity of obesity is conceptualised on the one hand, and for the practices promoted by the GROS on the other. Different models to analyse and predict the behaviour of complex systems are more or less able to accommodate internal complexity and the regulation of variation in input from outside the regulated system. This lead to a break between what has been referred to as first- and second-order cybernetics. First-order cybernetics, of the kind associated with Bateson (1972), sought to describe the total patterns that connect the elements of a system through loops of negative and positive feedback. It adopted a putative distinction between the inside and the outside of the system and aimed to provide an overarching, integrated perspective that assumed the totality or wholeness of the system. By contrast, theorists of second-order cybernetics, most notably Maturana and Varela (1992), aimed to bypass the “geography of inner versus outer” (p. 172). For Maturana and Varela, the looping that occurs in complex systems is always contingent on the observer. While first-order cybernetic systems are conceptualised as closed, total systems, second-order cybernetics posits a circular looping system more aptly represented by the image of the Mobius strip. Maturana and Varela (1992) propose to think the distinction between first- and second-order cybernetics in terms of a contrast between an integrated totality regulating the smaller parts and “an unruly conversational interaction: the very presence of this unruliness allows a cognitive moment to come into being according to the system's constitution and history” (p. 336). In such systems, referred to as ‘autopoeitic', the relationship between an organisation (or system) and its elements (or structure) is open-ended but not random. The elements in the system are neither just analytical constructs nor ontological: their existence is a factor of the reference point from which they are described. So while first-order cybernetics placed an emphasis on systemic homoeostasis, second-order cybernetics is based on the idea that it is not possible to see the totality from any particular point of view.

To which order of cybernetics do the feedback mechanisms mobilised in the obesity literature belong? Clearly, there are important differences within the scientific fields engaged in these debates. Most of the literature reviewed for this article provides a fairly closed and integrated model of regulation in which the biological system analysed (in rats or humans) and its – for the most part lab – environment are presented as ontologically distinct. Nevertheless, in their analysis of the cybernetics of body weight regulation, Fricke *et al* (2006) examine the role of systems thinking in analysing the molecular aspects of homoeostatic control of food intake. Their conclusion is worth citing at length for it marks a clear break with the tenor of much of the literature on homoeostatic regulation:

The attribution of control elements to biological structures always contains the problem of semantic simplification, because the biological structure is reduced to a singular meaning. The same biological structure can be a controlled variable in a certain feedback loop and a manipulated variable in a different loop. Therefore, feedback loops might be organized in chains, where elements are parts of different loops in different functions. (Fricke *et al*, 2006, p. 171)

An important question for future work in this area – both for scientific research and the examination of its policy implications – concerns the kinds of models that are put to work to conceptualise shifts in the way environments and bodies interact through ‘the sensorial.' Does the rise of ‘hedonism' and renewed attention to ‘addiction' in obesity science simply signal a historic shift in the food system, or does it also reflect a shift in epistemological understandings of the processes of appetite regulation? To what extent do first- or second-order cybernetic models actually influence the way research is carried out in this field? What are the genealogies of the models used in the different domains of obesity science and how do they come to impose themselves in the academic practices of different scientific communities? What political and conceptual implications do these different models have for the way we think about eating, treat its disorders and regulate the food system? In practice, the GROS method is messy. The interactions between sensations, food cues, desires, fears, habits and so on that it re-sensitizes eaters to are not neatly classifiable according to a topology of inside or outside, input or output. The method that has been drawn up experimentally from work with patients aims to return eaters to liking, rather than being driven by wanting (see Finlayson and Dalton, 2012).

## Conclusion

If obesity involves the biological defence of an elevated level of body fat, as current evidence suggests, advice to simply “eat less, move more” cannot be expected to remedy the problem. This is because interventions that reduce body fat stores without a corresponding decrease in the defended level of fat mass elicit compensatory responses that promote the recovery of lost fat and are difficult to consciously override. (Guyenet and Schwartz, 2012)

Public health initiatives aiming to curb the rising rates of obesity are often still implicitly based on the idea that controlling weight is a matter of willpower and choice. In this model, people are given information and are expected to understand it and change their behaviour in accord. Little recognition is given to the fact that there are a series of factors, ranging from the structural to the neurophysiological (themselves intrinsically tied up with global economic factors), that intercede in the neat progression that would lead from the provision of health information to changed eating behaviour.

In this article, I have mobilised scientific literature on the question of appetite regulation in order to reveal the models that are used to think about the complex interplay between foods, bodies and their environments. What has captured my attention is the way in which the different findings emerging from the fields of adiposity research or the science of appetite reconfigure the relative balance of agency between foods and eaters. Here foods are seen to subvert eaters' will by exerting certain influences on them that are beyond the realm of rational control. The intimate encounter between foods and eaters is not accidental or incidental but the product of fine-tuning between food production and homoeostatic and hedonic pathways of food intake regulation. This opens the question of the implications, for public health, of recognising the ways in which food may have agency over eaters. While industry has long understood and capitalised upon this, public health has been slower to do so.

I have grappled with two rather different sets of material effects in this article. The first concerns the material effects of foods (or fat, as stocked in adipose tissue) on eaters. The second concerns a political economy notion of materialism as it relates to the role of the food industry in both driving the first set of material effects and subsidising efforts to seek out solutions to the problems generated. Examining the way the science of sensoriality, food intake regulation and appetite control is mobilised in public health strategies targeting *le comportement alimentaire* (alimentary health behaviours) provides a vantage point onto some of the ways politics and metabolism collide and into the complex effects of scale that eating engages. What is striking is the recurrent play on a balance between reflective, information-based approaches understood to relate directly to people's will and agency and a growing consensus around the idea that food choices are in fact largely guided by affective, physiological, endocrine or otherwise unconscious mechanisms. This raises questions as to the kinds of initiatives that can be effectively mobilised to target what – notwithstanding the controversy surrounding the epidemiological claims regarding ‘overnutrition' – continues to be presented as a major public health priority.

## Footnotes

1 There is much to be said about the way industry has seized this notion of ‘pleasure' and the anti-dieting sentiment (notably through the industry support groups such as GROS receive). To give but one example, France's Weight Watchers launched a shock campaign provocatively titled “Stop Dieting! Relearn how to eat!” In January 2011 onwards, huge billboards appeared across France with provocative and highly sexualised images of voracious women's mouths bursting with all sorts of foods, from fries to sweets to broccoli.

2 This was corroborated by a report by the French National Agency of Food Security, Environment and Work (ANSES, 2010) on the physical and psychological risks of weight-loss dietary practices. The 10 most practised diets were evaluated for their risks for vital organ functioning, nutrient deficiencies and impact on food intake regulation mechanisms. The most damning aspect of the report concerns weight regain, estimated at 80 per cent of subjects at 1 year and higher beyond. This idea is not new to GROS, who has built its action around an anti-diet stance for over 10 years. However, the publication of the report lent support to a more diffuse anti-dieting sentiment arising from uncertainty about both the efficiency of diets and their long-term effects on health.

3 This faculty of bodily perception includes skin senses and proprioception of the internal organs.

4 On this topic, see Mann *et al* (2011).

5 This distinction between rational, will-governed behaviour and emotional, irrational or pleasure-driven behaviour finds parallels in public health approaches to the consumption of harmful substances that I do not address here but that would merit greater attention (see, for example, Greco, 1993, or Valverde, 1997).

6 Although she is referring to a discursive production concerning the loss of senses for ‘us' and a turn to the sensoriality of the ‘Other' in anthropology.

7 French distinguishes between ‘rassasiement' and ‘satiété'. The first concerns the decrease in hunger signals during a meal; it takes place roughly 15–20 min after meal initiation. The second occurs just after the first and is the period during which hunger is no longer experienced.

8 While not denying the complex processes of evolution that link the metabolic process at work in human bodies today to those of their prehistoric predecessors, my point is simply that such self-evident conclusions appear repeatedly in neurobiology or endocrinology articles without any reference to published research on these questions. Past environments are presented as homogenously poor in nutriments without any recognition of the vast diversity of environments in which humans have lived, thrived and evolved.

9 Fischler (2011) proposes that the French have been “protected” against obesity because they spend more time eating, and eating together, than the British or Americans, a fact that increases the chances of pleasure and satiety.
